# Deriving High-Energy-Density Polymeric Nitrogen N_10_ from the Host–Guest ArN_10_ Compound

**DOI:** 10.3390/nano15030249

**Published:** 2025-02-06

**Authors:** Lulu Liu, Jiacheng Qi, Dinghui Wang, Jie Yuan, Difen Shi, Zhigang Xiong, Ting Ye, Yubei Cai, Lei Zhang

**Affiliations:** 1 School of Electronic Engineering, Nanjing Xiaozhuang University, Nanjing 211171, China; 2 National Laboratory of Solid State Microstructures & Collaborative Innovation Center of Advanced Microstructures, School of Physics, Nanjing University, Nanjing 210093, China; 3 School of Materials Science and Physics, China University of Mining and Technology, Xuzhou 221116, China

**Keywords:** high-energy-density materials, first-principles calculations, polymeric nitrogen N_10_, robust stability

## Abstract

Discovering stable polymeric nitrogen phases and exploring their properties are crucial for energy storage and conversion, garnering significant attention. In this study, we investigate the formation possibility of a stable compound between Ar and N_2_ through ab initio calculations under low-pressure conditions (0–100 GPa). The novel super nitride, *Imm*2 ArN_10,_ is designed to demonstrate robust thermodynamic stability under high pressures (91 GPa) and showcase the unique host–guest structure, in which guest atoms (Ar) are trapped inside the host polymeric N_10_. Significantly, given the weak interaction between Ar and N atoms and a channel parallel to the *c*-crystallographic axis in ArN_10_, we propose a novel method to stabilize the previously unknown polymeric nitrogen structure, *Imm*2-N_10_, by removing the guest argon atoms from the natural channels of ArN_10_. *Imm*2 ArN_10_ and N_10_ are thermodynamically and dynamically stable, with energy densities of 9.1 kJ g^−1^ and 12.3 kJ g^−1^, respectively—more than twice that of TNT. Additionally, ArN_10_ and N_10_ stand out as leading green energetic materials, boasting a superior explosion velocity of 17.56 km s^−1^ and a detonation pressure of 1712 kbar, surpassing that of TNT. These findings significantly impact on the creation of pure nitrogen frameworks through chemical reactions involving inert elements under high pressure.

## 1. Introduction

In recent years, the significance of high-energy-density materials (HEDMs) has substantially grown within diverse energy-centric sectors, such as modern industry, civil engineering, weaponry, and space exploration [1,2]. To date, this surge in importance has consistently stimulated the interest of scholars specializing in condensed matter physics, chemistry, and materials science [3]. For example, cyclo-1,3,5-trimethylene-2,4,6-trinitamine (RDX) and 2,4,6-trinitrotoluene (TNT) have been developed in the past two centuries [4] as traditional energetic materials, nowadays generally utilized in both civilian and military applications. While the current explosives are indeed effective, ongoing research endeavors to focus on advancing the creation of novel HEDMs characterized by enhanced properties, environmental friendliness, and a higher energy density.

Polymeric nitrogen has emerged as the center of substantial interest due to its promising properties as an innovative HEDM [5], with the ability to store and release substantial amounts of energy through the rearrangement of bonds within its nitrogen molecules [6]. Upon the transformation of N_2_ into single- or double-bonded polymeric nitrogen crystals, there arises a crystalline phase characterized by an abundance of polymerized nitrogen, exhibiting a metastable state in comparison to its triply bonded counterparts. The energy released during this transformation has the potential to exceed that of any other known non-nuclear explosion, attributable to the significant energy disparity between a molecular entity of N_2_ with single bonds (N-N, ~160 kJ mol^−1^) and those with triple bonds (N≡N, 1 MJ mol^−1^) [7]. Moreover, polynitrogens are environmentally friendly due to their final product being nitrogen. Unlike traditional energetic materials, polymeric nitrogen exhibits notable advantages, boasting an energy density exceeding that of TNT by three times and yielding environmentally benign by-products (N_2_) [8]. Conversely, a nitrogen–nitrogen triple bond, recognized as one of the most robust chemical bonds, can transform its triple bond into single bonds, requiring a large amount of energy. Therefore, the substantial energy disparity between an N-N and an N≡N poses a formidable obstacle to the synthesis of polymeric nitrogen under ambient conditions [9].

As a result, the question of how to obtain singly bonded polymeric nitrogen is a crucial problem that urgently needs to be addressed. Typically, applying pressure to modify nitrogen’s chemical bonding characteristics has been demonstrated to be a valid method for stabilizing polymeric nitrogen phases [10,11,12]. Notably, the cubic gauche nitrogen (cg-N) structure has been successfully synthesized in experimental conditions characterized by an extremely high temperature and pressure (2000 K, 110 GPa) by Eremets et al. [13,14,15]. It is anticipated that cg-N will demonstrate an energy density surpassing that of the most potent energetic materials by a factor of more than three [13,16,17]. However, the ultrahigh pressure taken to stabilize it [18,19] diminishes its possibility of practical applications. Several new polymeric nitrogen forms of N_6_ [20], N_8_ [21], and N_10_ [22] with N-N and N=N have also been predicted by recent studies. These materials exhibit stability solely under high-pressure conditions and are thermally metastable down to ambient conditions suggested by dynamics calculations. On the other hand, it has not been experimentally proven, representing a huge challenge to preserve polymeric nitrogen compounds at ambient temperature and pressure. However, the addition of non-nitrogen elements can reduce the pressure during synthesis, providing an alternative route to synthesize materials with N-N or N=N. For example, attention has been concentrated on N_2_H [23], t-N [6], CrN [24], cyclo-N_5_^–^ [25], and ZnN [26]. Through compressing NaN_3_ with N_2_, the pressure required to synthesize the cg-N can fall to 50 GPa in pure N_2_ [19]. The lower stabilization pressure of polynitrogen compounds might result from the chemical precompression caused by other elements [27], the diversity of N-N bonds beyond the N_2_ molecule [28], or the additional chemical bonding between N and other elements [29]. Regardless, the bonds formed between non-nitrogen elements and nitrogen elements diminish the nitrogen bond content of the polymerized nitrogen, potentially resulting in a reduced energy density of the material [23].

Therefore, the choice of the inert element is predicated on its non-reactivity with the nitrogen element, ensuring the absence of bonding between them. The same idea has been validated in several previous studies. A van der Waals Xe-N_2_ compound has been synthesized [30,31], indicating that noble gas atoms can form compounds with nitrogen. The route to obtain stable high-energy-density xenon nitride at high pressures was raised by Peng et al. [32], implying that noble gases can promote the formation of pure polymeric nitrogen from nitrogen under high pressure. This discovery points us in a new direction for our research. However, our study turns its attention to another noble gas, argon. As an essential member of the inert elements and as the heaviest noble gas, argon, whose valence electrons are shielded by core electrons and, thus, are less strongly bound, could form stable molecules [33]. Furthermore, the incorporation of noble gases into the nitrogen system has the potential to substantially modify long-range Coulomb interactions, a phenomenon which can reduce the synthesis pressure necessary for the formation of polynitrogen compounds [34,35].

Notably, nitrogen constitutes the predominant gas in the Earth’s atmosphere, and argon, the third largest component of the atmosphere, is also widely present in the Earth’s crust [36,37]. Multiple structures of pure N_10_ have been investigated. For example, there are pure caged-type N_10_ clusters [38] and N_10_ chains [22]. However, the studied nitrogen clusters N_10_ are unstable. An all-nitrogen molecular crystal composed solely of bispentazole N_10_ molecules with an extremely high energy density (5.5 kJ g^−1^) has been predicted [39], along with the N_5_^+^N_5_^−^ salt [40,41]. Regarding the argon–nitrogen system, the argon–nitrogen phase diagram [42,43] has been studied. However, in the Ar-N system, nitrogen exists in the form of nitrogen molecules and fails to form a nitrogen framework structure. As a whole, the absence of argon nitrides significantly hinders the comprehension of their characteristics and prospective applications. Consequently, conducting a comprehensive investigation into the potential reactivity between Ar and N_2_ under extreme conditions holds immense importance for facilitating the discovery and application of HEDMs [44].

In this paper, we present ab initio structure prediction calculations covering a wide composition range of the argon–nitrogen (Ar-N) system. Within the experiment, we identify *Imm*2 ArN_10_ as a stable compound under 100 GPa. We investigate the three-dimensional structure of this material under high pressure and discover many excellent properties of this material through calculations of band structure and phonon. This polynitrogen consists of a pure polymerized nitrogen framework that incorporates interstitial argon. Due to the weak interaction between the interstitial argon and the polymerized nitrogen framework, our findings indicate that the nitrogen framework is maintained at 100 GPa even after the extraction of argon, resulting in the formation of a novel, pure nitrogen polymeric solid. This nitrogen polymeric solid, noted as *Imm*2 N_10_, is dynamically stable at 100 GPa. Likewise, through the analysis of band structure, phonon, and partial charge density, *Imm*2 N_10_ may be a potential new material with semiconductivity and high energy density.

## 2. Computational Details

We searched for crystal structures using an unbiased swarm intelligent structure prediction method, as performed in the CALYPSO code [45,46]. This approach has achieved success in uncovering intricate structures across numerous systems [47,48,49,50,51]. For structural optimizations and calculations of electronic properties, we utilized density functional theory. Specifically, we adopted the Perdew–Burke–Ernzerhof (PBE) functional [52] within the generalized gradient approximation (GGA) [53], which is implemented in the VASP 5.4.4 code [54]. Pseudopotentials were utilized within the projector augmented wave (PAW) [55]. For argon and nitrogen atoms, the valence electrons considered were 3s^2^ 3p^6^ and 2s^2^ 2p^3^, respectively. A kinetic energy cutoff of 700 eV and a Monkhorst–Pack scheme [56] with a *k*-point grid of 2π × 0.025 Å^−1^ were adopted to ensure that total energy calculations converged to less than 1 meV per atom. We employed the finite displacement method from PHONOPY, integrated with VASP. It displaces atoms from equilibrium, calculates forces, and constructs a dynamical matrix to get phonon frequencies and eigenvectors. We first optimized the atomic positions and lattice parameters of the structure using VASP with the PAW method and the PBE functional. Then, to account for long-range interactions, a 2 × 2 × 2 (80 atoms) supercell was constructed along the a-, b-, and c-axis. Finally, within this supercell, the phonon spectra were calculated using the finite displacement approach [57] implemented in the Phonopy code [58]. Van der Waals interactions were also considered during the structure optimization using the DFT-D3 functional [59] and the AIMD within the NpT ensemble with a Langevin thermostat [60]. Specifically, for the ArN_10_, the simulations were carried out in 2 × 2 × 2 supercells containing 176 atoms, while, for the N_10_, the 2 × 2 × 2 supercells consisted of 160 atoms. We used a step size of 0.5 fs during the molecular dynamics simulation. The extent of electron localization was assessed by applying the electron localization function (ELF) [61]. To quantitatively depict the chemical bonding features, crystal orbital Hamilton populations (COHP) [62], incorporated in the LOBSTER program [63], were employed. The non-covalent interactions (NCIs) present in molecular structures were scrutinized using the CRITIC2 code [64,65]. The steric non-covalent interactions were made visible with the help of the Visual Molecular Dynamics software [66].

## 3. Results and Discussions

### 3.1. Thermodynamic Stability

To obtain the thermodynamically stable Ar-N phases, our study mainly focused on three key aspects. We explored the impact of pressure by searching structures at 0, 50, and 100 GPa. The Ar:N atomic ratio was systematically varied, and we focused on nitrogen-rich ArN_x_ (*x* = 1–10) compounds. The formation enthalpy was calculated, and a convex hull diagram was used to evaluate material stability.

We calculated the formation enthalpies of each ArN*_x_* structure with the lowest enthalpy at the corresponding pressure, where the formation enthalpy Δ*H* is defined as:

ΔH*_f_* = [*H*(ArN*_x_*) − *H*(Ar) − x/2*H*(N*_2_*)]/(1 + *x*) (1)

**Figure 1:**
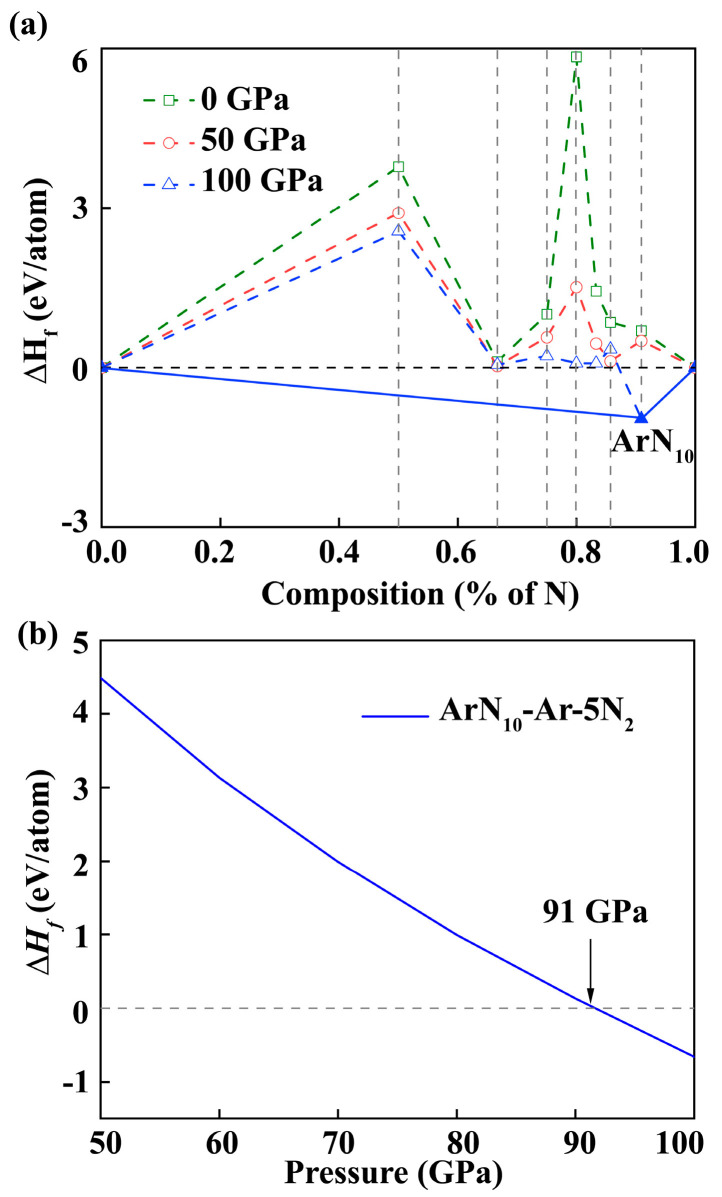
(**a**) Convex hulls for formation enthalpies per atom (Δ*H_f_*, concerning pure argon and nitrogen) are built under different pressures of 0, 50, and 100 GPa. (**b**) Enthalpy difference of *Imm*2 ArN_10_ related to the mixtures of Ar and N_2_ at 50–100 GPa.

Subsequently, the derived Δ*H_f_* for each ArN_x_ at a specified pressure were utilized to construct the convex hull. Compounds with thermodynamic stability were situated on the convex hull (solid line), while compounds positioned along the dotted lines were considered energetically unstable. Based on the convex hulls presented in Figure 1a, we identified a stable nitrogen-rich phase, designated as *Imm*2 ArN_10_. At pressures of 0 and 50 GPa, there were no compounds exhibiting negative formation enthalpies in relation to mixtures of *Fm*-3*m* Ar, *Pa*-3, and *P*4_1_2_1_2 N_2_ [67,68]. With an increase in pressure, for the stoichiometry of ArN_10_ with the highest nitrogen content, the enthalpy of formation at 100 GPa was negative, indicating that this structure is thermodynamically stable. Further calculations showed that ArN_10_ becomes thermodynamically stable at 91 GPa, as shown in Figure 1b. This is the first time we have found a stable compound in the Ar-N system.

### 3.2. Crystal Structures

**Figure 2:**
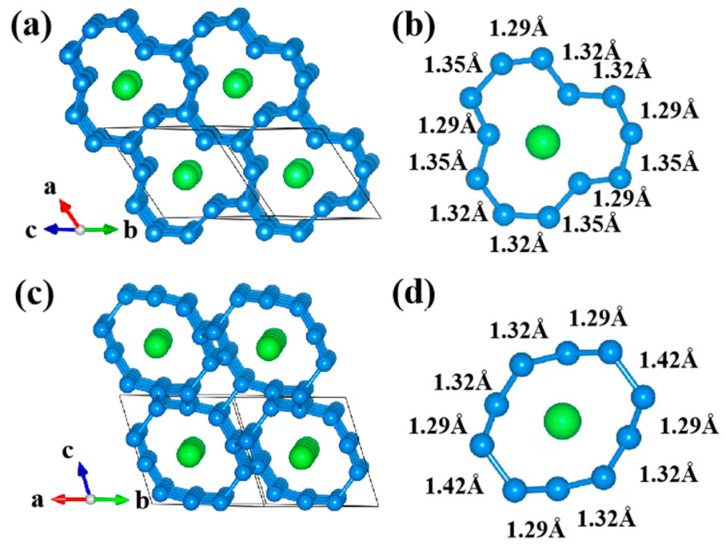
(**a**) The unit cell of the *Imm*2 structure, from the perspectives of (0.726305, 0.631516, 0.271418). (**b**) Ar and the surrounding 12 N atoms. (**c**) The unit cell of the structure in another sight, from the perspectives of (0.013440, 0.001710, 0.999909). (**d**) Ar and the surrounding 10 N atoms. The relatively large green spheres represent argon (Ar) atoms, while the relatively small blue spheres represent nitrogen (N) atoms.

ArN_10_ is a triclinic structural compound with a space group of *Imm*2. ArN_10_ is arranged in clusters (Figure 2a,b), forming polygonal channels that are symmetric and large enough to accommodate argon atoms, thus forming a host–guest structure. The structure of ArN_10_ can be observed from different angles in a layered and parallel arrangement. Argon atoms are situated at the center of a cyclic structure. In terms of atomic bonding, Ar atoms are scattered in the framework of N atoms, and the minimum argon–nitrogen distance is 2.34 Å, which is beyond the range of chemical bonding, signifying that the formation of an argon–nitrogen bond is highly unlikely.

*Imm*2 ArN_10_ shows two types of nitrogen atomic structure: N-N decacyclic and N-N dodecacyclic, both of which are in the shape of zig-zag chains. The ten-membered and twelve-membered rings are systematically stacked and interconnected in three-dimensional space, resulting in a periodic large framework structure that forms channels. Ar is surrounded by 12 N atoms (Figure 2b), with the nearest-neighbor separation measuring 2.34 Å and the next-nearest-neighbor separation measuring 2.89 Å. The N-N bond is from 1.29 to 1.35 Å, situated between the length of a single bond (1.45 Å) and a double bond (1.20 Å), revealing peculiar bonding properties. From an alternative standpoint, within the decacyclic ring (Figure 2c,d), the internuclear distances of nitrogen–nitrogen pairs range from 1.29 to 1.43 Å, and the bond angles span from 116.96 to 124.56°. This situation is consistent with the sp^3^ hybridization. The variations in bond lengths and the number of symmetry planes between the ten-membered and twelve-membered rings lead to distinct stacking configurations which, in turn, have differing impacts on the formation of the large framework and the channels. The symmetry planes of the ten-membered rings facilitate a regular arrangement along the c-axis, thereby promoting a symmetrical and orderly channel structure. In contrast to the twelve-membered ring, the ten-membered nitrogen ring has two symmetrical planes. The average bond length (1.32 Å) can significantly influence the energy density. This suggests that nitrogen-rich *Imm*2 ArN_10_ has great potential as a promising candidate for HEDMs. Conversely, the unique characteristics of the twelve-membered rings can alter the local shape and spatial distribution of the channels when co-constructing the framework with the ten-membered rings, potentially resulting in specific curvatures or expanded regions. Therefore, if one wants to extract Ar atoms, it should be done along the channels of ten-membered rings (*c*-crystallographic axis). The Ar atom is removed, and the remaining pure polymeric nitrogen still has a space group of *Imm*2 at 0–100 GPa. Such a structural character is conducive to separating Ar atoms from the channel without destroying the nitrogen frame structure, similar to the obtained Si, Ge-clathrates, and H_2_O [69,70]. The crystal structural information is shown in Supplementary Tables S1 and S2.

### 3.3. The Dynamic and Thermal Stability

**Figure 3:**
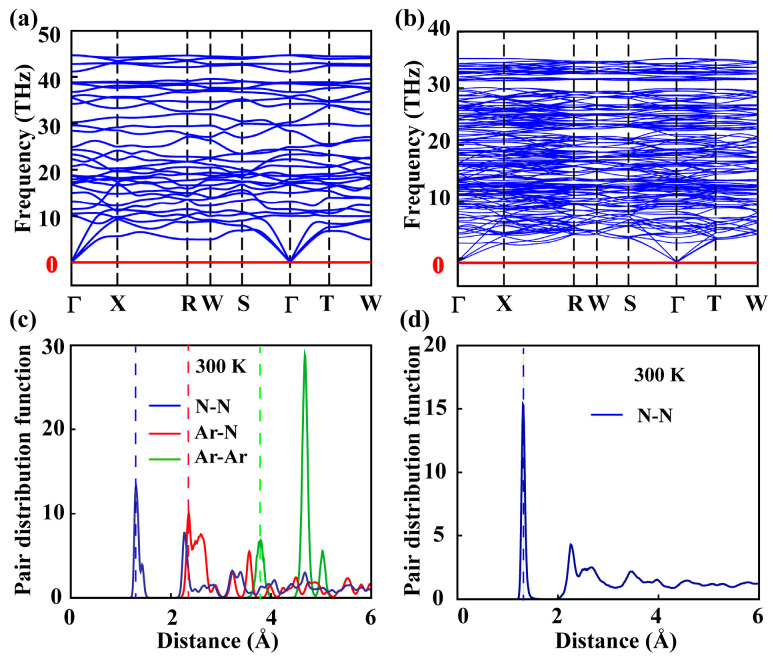
Phonon dispersion of (**a**) *Imm*2 ArN_10_ and (**b**) *Imm*2 N_10_ at 100 GPa. Among them, the red horizontal line represents the reference line of 0 HZ. (**c**,**d**) Pair distribution functions of AIMD simulations of *Imm*2 ArN_10_ and *Imm*2 N_10_ at 300 K, wherein the vertical dashed lines represent the nearest atomic distances of N-N, Ar-N, and Ar-Ar of the original structure.

The phonon spectra were computed to verify the dynamic stability of ArN_10_ and N_10_. The dynamic stability of materials can be assessed by examining the phonon spectrum for the presence of imaginary frequencies. The phonon spectrum of *Imm*2 ArN_10_ was calculated and is shown in Figure 3a. It turns out that the majority of the acoustic vibration modes were predominantly localized within the frequency range of 0 to 11 THz. The absence of imaginary frequency was found throughout the entire Brillouin zone, a phenomenon which indicates the dynamic stability of *Imm*2 ArN_10_. As a previously unexplored material, we find that pure polymeric nitrogen *Imm*2 N_10_, through phonon spectrum calculations, is still dynamically stable (Figure 3b).

**Figure 4:**
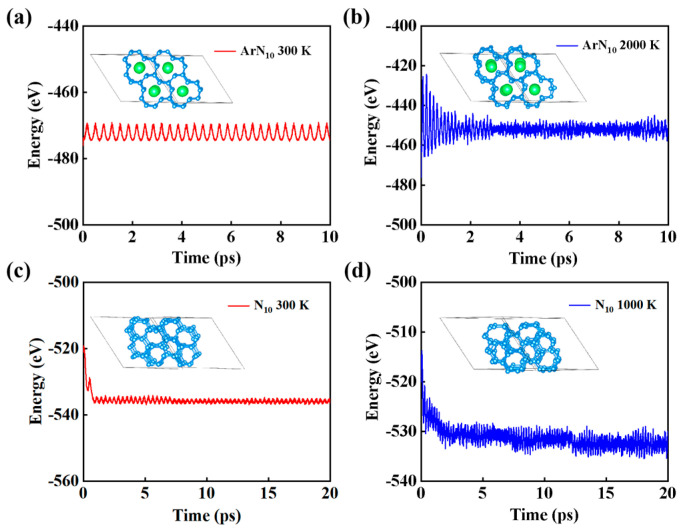
Molecular dynamics simulation of (**a**) *Imm*2 ArN_10_ at 300 K, (**b**) *Imm*2 ArN_10_ at 2000 K, (**c**) *Imm*2 ArN_10_ at 300 K, (**d**) and *Imm*2 ArN_10_ at 1000 K. The structure of each graph is the final structure.

The radial distribution functions (RDFs) were utilized to examine the thermal stability of *Imm*2 ArN_10_ and *Imm*2 N_10_. Specifically, the simulation for *Imm*2 ArN_10_ ran for 10 ps at 300 K, while that for *Imm*2 ArN_10_ lasted for 20 ps at the same temperature of 300 K. The total energy of these structures exhibited minimal variation over a duration of 10 ps with a 0.5 fs step, as illustrated in Figure 4, ensuring that the total energy calculations converged at the considered step. Therefore, the equilibrium structures were derived from the final stage of AIMD simulations, unambiguously demonstrating the validity of molecular dynamics simulations, and the polymeric nitrogen demonstrated a high degree of stability during the simulations conducted at 300 K, as illustrated in Figure 4a. Notably, the prominent peaks located on the left side of each line correspond to the closest N-N, Ar-N, and Ar-Ar distances, respectively, as illustrated in Figure 3c. For ArN_10_, the first sharp peak of RDF occurred at approximately 1.32 Å, which aligns closely with the shortest N–N bond length, observed in the N_2_ dimer in the original structure, as represented by the vertical dashed line. The initial pronounced peak of the Ar–N interaction occurred at approximately 2.36 Å, aligning closely with the minimum distance observed between the argon and nitrogen atoms in the original configuration, and this observation implies thermal stability within the structure. Moreover, the fact that the initial sharp peak of *Imm*2 N_10_ was situated precisely at 1.32 Å, aligning with the peak of *Imm*2 N_10_, serves as additional evidence for its thermal stability, as illustrated in Figure 3d. In addition, we found that *Imm*2 ArN_10_ and *Imm*2 N_10_ are thermodynamically stable at higher temperatures (e.g., 2000 K and 1000 K) (as shown in Figure S1). Overall, the equilibrium structure exhibited minimal changes in the closest distances among atoms when compared to the original structure, indicating its thermal stability.

### 3.4. Electronic Properties and Bonding Characteristics

**Figure 5:**
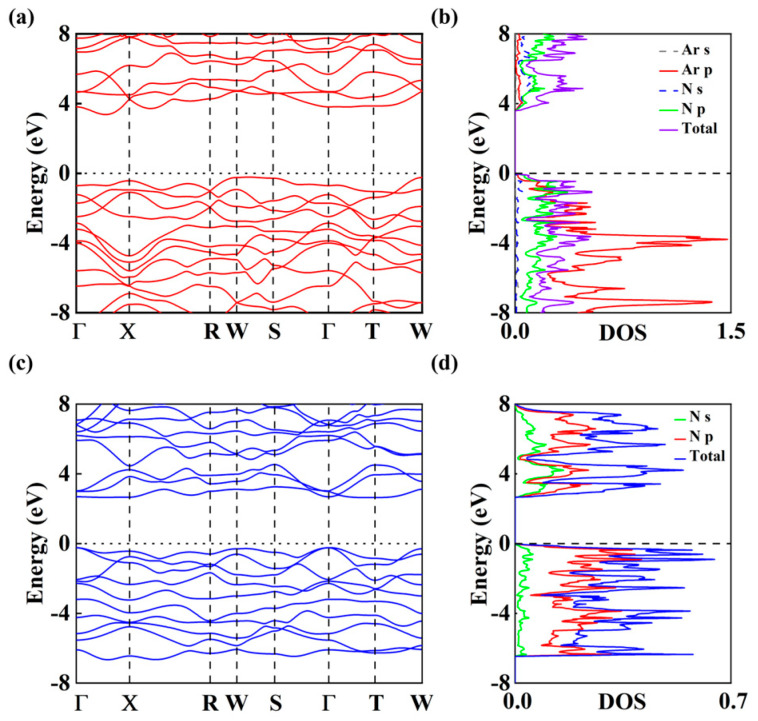
(**a**) Electronic band structure of *Imm*2 ArN_10_, (**b**) density of states of *Imm*2 ArN_10_, (**c**) electronic band structure of *Imm*2 N_10_, (**d**) density of states of *Imm*2 N_10_. All the results shown in (**a**–**d**) are based on PBE. A direct band gap is shown by a red arrow.

**Figure 6:**
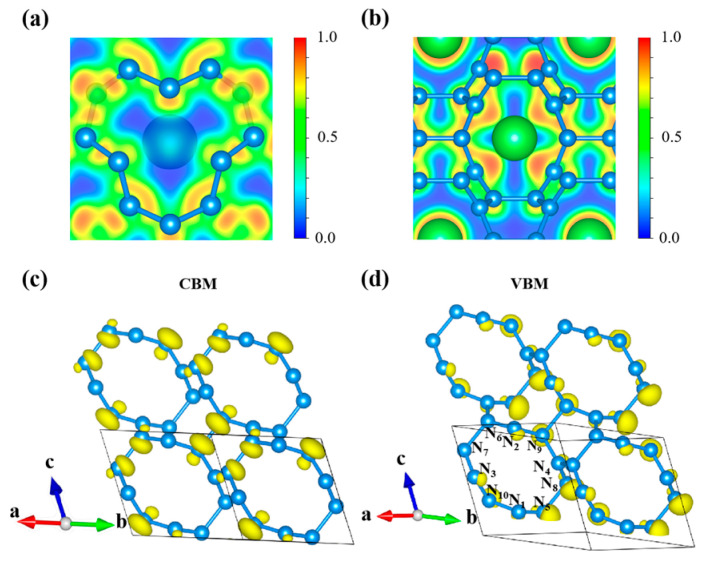
(**a**,**b**) The electronic localization function (ELF) of *Imm*2 ArN_10_ at 100 GPa from the perspectives of (−0.84, 0.04, −0.55) and (−0.19, 0.09, −0.98), (**c**) partial charge density corresponding to frontier states in the conduction band, and (**d**) valence band of *Imm*2 N_10_.

To gain a more in-depth understanding of the electronic properties of the predicted Ar-N compound, its electronic band structures and electronic density of states (DOS) were calculated, as shown in Figure 5a,b. The results indicate that, for ArN_10_, the conduction band minimum lies between the Γ and M paths, while the valence band maximum is located at the position of the high-symmetry path M. The *Imm*2 phase of ArN_10_ exhibits a band gap value of 3.58 eV at a pressure of 100 GPa, a value which classifies it as a semiconductor [71]. Moreover, within the pressure range of 0 to 100 GPa, the band gap gradually increases with the increase in pressure, with the band gap size ranging from 0 to 3.58 eV. This also shows that the band gap of ArN_10_ does not disappear as the pressure increases (Figure S3). Different colors in the DOS plots indicate the electronic contributions of nitrogen atoms from different orbitals. The N 2p orbital electrons contribute significantly to the energy density of states below the surrounding Fermi level. The DOS shows strong N-P hybridization, pointing to covalent bonds in the N_10_ and N_12_ macrocycles, a finding which is consistent with our above structure analysis of *Imm*2 ArN_10_ (Figure 5d). We also investigated the electronic properties of *Imm*2 N_10._ Figure 5c,d display the electronic band structure and the corresponding DOS. Using the PBE function, we found that *Imm*2 N_10_ is a direct semiconductor with a band gap of 2.87 eV at a pressure of 100 GPa, as depicted in Figure 6c.

The nature of chemical bonding significantly influences the stability of polynitrogen structures. Covalent bonds have higher ELF values (near 1.0). As shown in Figure 5a,b, the calculated electronic localization functions of ArN_10_ at 100 GPa indicate that there are strong covalent bonds among the nitrogen atoms in the N_10_ and N_12_ macrocyclic frameworks. The maximum of the ELF in ArN_10_ appears as lobes extending outward from the nitrogen atoms, a phenomenon which is indicative of lone pairs. For the ELF of *Imm*2 ArN_10_ in Figure 5a, the N atoms hybridize into sp^2^ and sp^3^ states. Conversely, the ELF values in the interstitial regions between the Ar and N atoms are close to 0. This indicates that there is no covalent bonding between the Ar and N_10_ rings.

To gain deeper insights into the electronic properties of nitrogen structures, the electron characters of the conduction band minimum (CBM) and the valence band maximum (VBM) of ArN10 were explored by partial charge density plotting (as shown in Figure S2). As shown in Figure 5c,d, both the CBM and VBM of *Imm*2 N_10_ are located at the Γ (0.0, 0.0, 0.0) point. The charge distribution in the VBM was found to surround N_9_ and N_10_ atoms and to appear as a spherical-like electron cloud. Something interesting took place during the transition from VBM to CBM. The charge distribution in the CBM became much different: the electrons from N_9_ and N_10_ underwent hybridization and formed a bowling ball with one large and one smaller end. Currently, the symmetry of the formation of the *π* bond is no longer satisfied, forming a *σ* bond to satisfy the maximum overlap of the orbitals. In this state, the N atoms are mainly connected to each other in the form of covalent bonds (N-N). The result can be further verified by detailed DOS plotting for different orbitals, shown in Figure 5d. Again, the lone electron pair of every N atom is not connected with the charge densities of the other atoms (Figure 6c,d).

To further reveal the relationship between the Ar and N atoms, we computed the ICOHP, which quantifies the binding interactions between two types of atoms. The -ICOHP of most strong N-N atomic pairs was greater than 9.6 eV pair^−1^, indicating strong bonding. However, the -ICOHP value for Ar–N atomic pairs was relatively low, less than 0.1 eV pair^−1^, indicating that there was no bond between Ar and N, a finding which is consistent with the above ELF analysis. The negatively projected COHP (-pCOHP) curve of the average N-N pair (Figure S4) shows that there were bonding and antibonding interactions between Ar and N at the energy level of −7.0 to 0 eV below the top of the valence band, but the -pCOHP was close to 0 above and far below the Fermi level.

Moreover, we found some interesting properties of *Imm*2 ArN_10_ after the calculations of Bader’s effective charges (Table S3). The results show that the electron transfer between the Ar atom and the N atom was minimal, only 0.031eV at 100 GPa and close to 0 eV at 0 GPa, while the electron transfer between Ar and the N_10_ ring was even smaller, about 0.003 eV, on average, at 100 GPa, a finding which can confirm that there is a non-bonded interaction between the Ar atom and the N_10_ ring by the partial charge density. The presented bond characteristic of *Imm*2 ArN_10_ is in good agreement with the condition of ELF.

Nevertheless, Ar is stabilized in the polymerized nitrogen framework; therefore, we calculated weak interactions, which play an important role in chemistry, biology, and material design, to analyze stability. Due to the weak electron localization, some analysis algorithms (such as the Bader’s charge transfer and ELF) cannot easily identify complex NCIs. Therefore, we used CRITIC2 codes to read the scalar fields generated by the crystal structure and VASP calculation results. In addition, the analysis of the NCIs of ArN_10_ supplemented the quantum chemical bonding information. The obvious sharp peak at ρ = 0.1 au indicates a strong non-bonded overlap located in the ArN_10_ structure (green contour in the lower row of Figure S5). There may be a weak interaction to stabilize the Ar atoms in the nitrogen framework contributing to the Ar atoms stabilizing the nitrogen gas into polymerized nitrogen crystals under high pressure. Considering the weakness of the interaction between Ar and the nitrogen atoms, we prove that it is also possible to remove Ar from the ArN_10_ structure to obtain N_10_.

### 3.5. Energy Density and Explosive Performance

To confirm the probability of the N-rich *Imm*2 ArN_10_ as a promising candidate for high-energy-density materials, we assessed the energy released from the decomposition of nitrides into gaseous Ar and N_2_. Additionally, the energy difference of 0.5 eV [72] during the transition from solid N_2_ to a gaseous state at 300 K was considered. The calculation results revealed that the energy density of the *Imm*2 ArN_10_ and *Imm*2 N_10_ structures is 9.1 kJ g^−1^ and 12.3 kJ g^−1^, respectively. These values are about five times that of TNT (1.6 kJ g^−1^) and roughly twice that of HMX (6.7 kJ g^−1^) [73,74].

The detonation properties of the *Imm*2 ArN_10_ structure are crucial metrics in assessing its viability as an HEDM [75]. These properties include the detonation velocity (*V_d_*) and the detonation pressure (*P_d_*), both of which provide insights into the material’s explosive power and performance. *V_d_* and *P_d_* can be estimated based on the Kamlet–Jacobs empirical equation [73], and their formulas are as follows:

*V_d_* = 1.01(*NM*^0.5^
*E_d_*^0.5^)^0.5^(1 + 1.30*p*) (2)

*P_d_ =* 1.58 *p*^2^
*NM*^0.5^
*E_d_*^0.5^ (3)

Herein, *N*, *M*, and *p* stand for the concentration of N_2_ in terms of moles per gram of explosive material (expressed as mol g^−1^), the molecular weight of the N_2_ gas (equivalent to 28.00 g mol^−1^), and the density (measured in g cm^−3^), respectively. In the above formulas, the units of the gravimetric chemical energy density (*E_d_*) should be converted to kJ Kg^−1^. To ensure calculation accuracy, the most significant figures were retained. We retained the most significant figures for *Imm*2 ArN_10_ and N_10_ which were about 9.1 kJ g^−1^ and 12.3 kJ g^−1^, respectively. It was estimated that the *V_d_* and *P_d_* of the *Imm*2 ArN_10_ phases can reach 17.56 km s^−1^ and 1712 kbar, respectively, and the *V_d_* and *P_d_* of the ArN_10_ can reach 22.35 km s^−1^ and 2832 kbar, respectively. These values are about two times and nine times higher than those of TNT (6.9 km s^−1^, 190 kbar), as well as being approximately two times and four times higher than those of HMX (9.1 km s^−1^, 393 kbar) [73,74]. The exceptional detonation performance exhibited by *Imm*2 ArN_10_ indicates its potential for use in high explosives. It is important to mention that the *V_d_* and *P_d_* of traditional energetic materials like TNT and HMX are typically determined through experimental measurements. More information on explosion performance is shown in Table S4 of the Supplementary Materials. However, with regard to *Imm*2 ArN_10_, its performance parameters are estimated using first-principles methods, without considering any energy losses that may occur during experiments. Therefore, it is likely that the actual detonation velocities and pressures of *Imm*2 ArN_10_ may be slightly lower than the calculated values.

## 4. Conclusions

Our comprehensive analysis of Ar-N compounds based on first principles and structure search techniques unveils a groundbreaking discovery, as we predict the successful synthesis of Ar-N compounds at lower pressures by introducing argon into the nitrogen system. Particularly noteworthy is our observation that *Imm*2 ArN_10_ demonstrates energetic stability above 91 GPa, a pressure threshold lower than that required for pure polynitrogen synthesis. The unique N_10_ ring, with high-symmetry polymeric chains, in *Imm*2 ArN_10_ determines distinctive chemical and physical characteristics. Furthermore, our exploration of the bonding characteristics and electronic properties of *Imm*2-ArN_10_ unveils the exceptional non-bonding interactions between the polymeric nitrogen and the central Ar atoms, such interactions stabilizing the guest atoms within the host framework. Consequently, we propose the removal of Ar from the natural channels of the ArN_10_ structure, leading to the formation of the distinct pure polymeric nitrogen structures of *Imm*2-N_10_. Additionally, phonon calculations and AIMD simulations indicate the dynamic and thermal stability of *Imm*2 ArN_10_ and *Imm*2 N_10_. Notably, the energy density of *Imm*2-ArN_10_ and *Imm*2-N_10_ is twice that of TNT. Our research plays a pivotal role in the exploration of new high-energy-density materials.

## Data Availability

The data that support the findings of this study are available from the corresponding author upon reasonable request.

## Supplementary Materials

The following supporting information can be downloaded at: https://www.mdpi.com/article/10.3390/nano15030249/s1, See the Supplemental Materials, which include the calculated equations for the detonation velocity and detonation pressure of TNT, the crystal structure prediction methods, the molecular dynamics simulation, and the pair distribution functions of the AIMD simulations of *Imm*2 ArN_10_ and *Imm*2 N_10_, the pressure-dependent band gap curves of *Imm*2 ArN_10_, the -pCOHP calculation for characterizing the chemical bonds of *Imm*2 ArN_10_, the two-dimensional (2D) plots of the RDG of *Imm*2 ArN_10_, the structural information of the predicted Ar-N compounds, the charge transfer in *Imm*2 ArN_10_ at selected pressures, the explosive performance, and the structural information of the predicted Ar-N compounds (vasp). More detailed parameters are provided in the Supplementary Materials [45,46,52,53,54,55,56,57,58,59,60,61,62,63,64,65,66].
