# Supplementary material for: Deriving High-Energy-Density Polymeric Nitrogen N10 from the Host–Guest ArN10 Compound
Source: Nanomaterials (Basel). 2025 Feb 6;15(3):249. doi: 10.3390/nano15030249 (PMC11820297; doi:10.3390/nano15030249)
Supplement: Supplementary file 1 [file nanomaterials-15-00249-s001.zip › nanomaterials-3437530-supplementary.pdf]

nanomaterials

Supporting Information

Deriving High-Energy-Density Polymeric Nitrogen N_{10} from the Host–Guest ArN_{10} Compound

Lulu Liu [1,2,*], Jiacheng Qi [1], Dinghui Wang [3], Jie Yuan [1], Difen Shi [1], Zhigang Xiong [1], Ting Ye [1], Yubei Cai [1] and Lei Zhang [1,*]

[1] School of Electronic Engineering, Nanjing Xiaozhuang University, Nanjing 211171, China
[2] National Laboratory of Solid State Microstructures & Collaborative Innovation Center of Advanced Microstructures, School of Physics, Nanjing University, Nanjing 210093, China
[3] School of Materials Science and Physics, China University of Mining and Technology, Xuzhou 221116, China
* Correspondence: liululu@njxzc.edu.cn (L.L.); zl_srd@njxzc.edu.cn (L.Z.)

Computational details

We searched for crystal structures using an unbiased swarm-intelligent structure prediction method as performed in the CALYPSO code [1,2]. Structural optimizations and electronic property calculations were performed using density functional theory within the Perdew-Burke-Ernzerhof (PBE) [3] of generalized gradient approximation (GGA) [4] as implemented in the VASP5.4.4 code code [5]. Pseudopotentials were employed within projector augmented wave (PAW) [6] method with $3s^2$ $3p^6$ and $2s^2$ $2p^3$ valence electrons for Ar and N atoms, respectively. A kinetic-energy cutoff of 700 eV and a Monkhorst-Pack scheme [7] with a *k*-point grid of $2\pi \times 0.025$ Å^{-1} were adopted were adopted to ensure that total energy calculations converged to less than 1 meV per atom. Phonon spectra calculations were carried out by using the finite displacement approach [8] as performed in the Phonopy code within the 2 × 2 × 2 (80 atoms) supercells [9]. The vdW interactions are also taken into consideration by using DFT-D3 functional [10]. Using AIMD within the *NpT* ensemble with a Langevin thermostat [11]. The AIMD simulations was carried out within the 2 × 2 × 2 (176 atoms) supercells for ArN_{10},. Electron localization function (ELF) was utilized to measure the degree of electron localization [12]. Crystal orbital Hamilton populations (COHP) [13] as implemented in the LOBSTER program [14] was used to quantitatively characterize the chemical bonding properties. The noncovalent interactions (NCIs) in molecular structures were analyzed by the CRITIC2 code [15,16]. The steric NCIs are visualized through Visual Molecular Dynamics software [17]. More detailed parameters are provided in the Supplemental Material.

The noncovalent interactions in molecular structures were analyzed by the CRITIC2 code. To determine the dynamical stability of predicted structures, phonon calculations were performed by using the finite displacement approach as implemented in the Phonopy code. The TNT is detonated under ideal conditions in the following way: $2C_7H_5N_3O_6 \rightarrow 3N_2$ (g) + 7CO (g) + $5H_2O$ (g) + 7C (s) + Energy. For HMX: $C_4H_8N_8O_8 \rightarrow 4N_2$ (g) + 4CO (g) + $4H_2O$ (g) + Energy

Here, we adopt the $P2_1/c$ $C_7H_5N_3O_6$, P21/c $C_4H_8N_8O_8$, α-nitrogen, $P2_12_12_1$ CO, $Cmc2_1$ H_2O, R-$3m$ graphite to calculate the explosive performance.

Supporting Figures

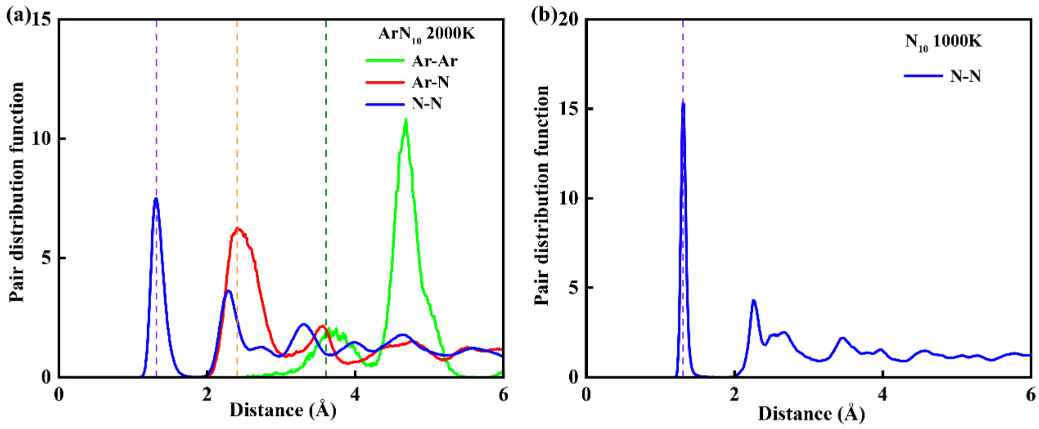

Figure S1. (a) Pair distribution functions of AIMD simulations of *Imm*2 ArN_{10} at 2000 K and (b) *Imm*2 ArN_{10} at 1000 K, wherein the vertical dashed lines represent the nearest atomic distances of N-N, Ar-N, and Ar-Ar of the original structure.

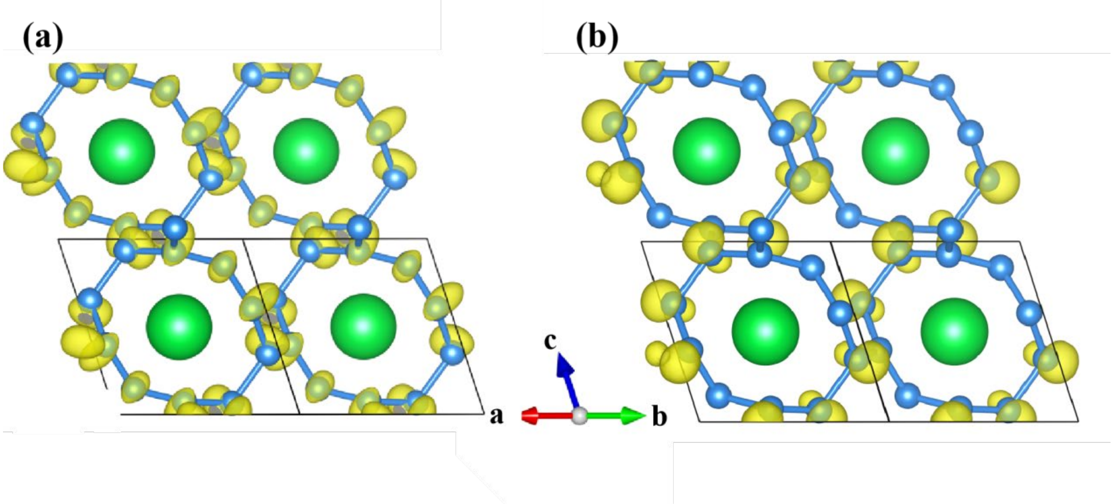

Figure S2. (a) partial charge density corresponding to frontier states in the conduction band, and (b) valence band of *Imm*2 ArN_{10}.

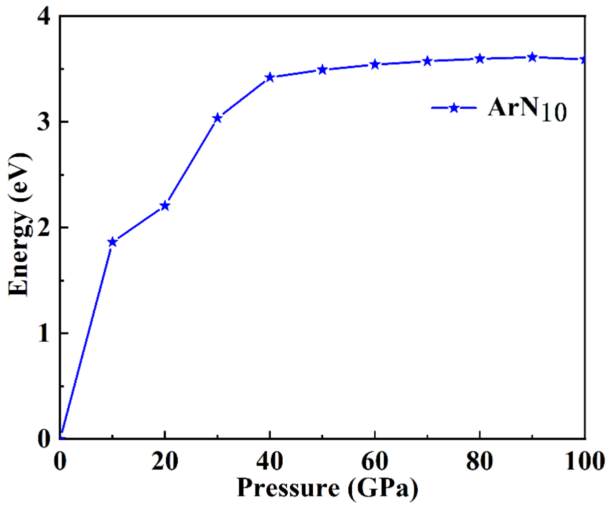

Figure S3. The pressure-dependent band gap curves at the PBE leve of *Imm*2 ArN_{10}.

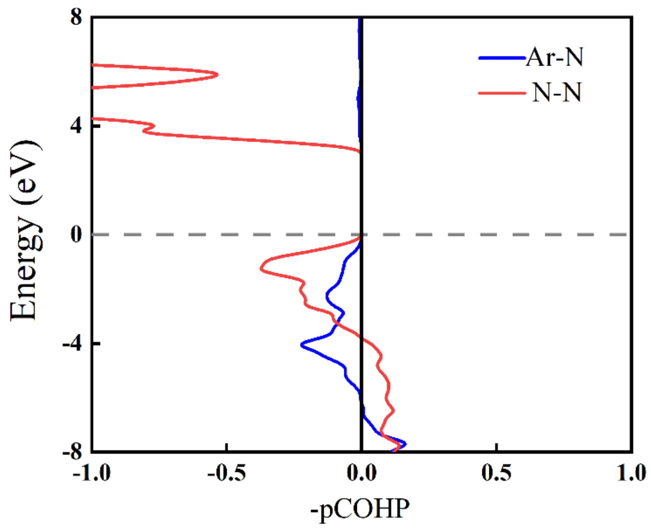

Figure S4. -pCOHP calculation for characterizing chemical bonds of *Imm*2 ArN_{10}.

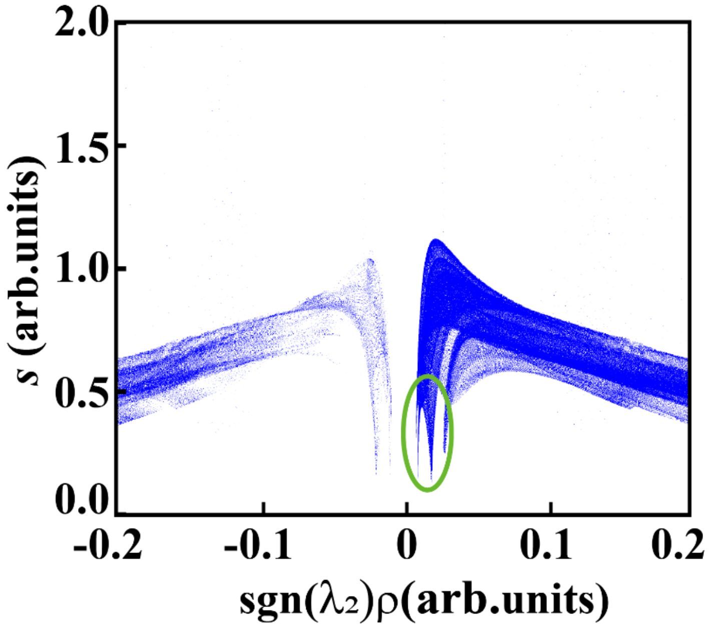

Figure S5. The 2D plots of RDG of *Imm*2 ArN_{10} versus the electron density multiplied by the sign of the second Hessian eigenvalue ($\lambda 2$).

Table S1. Structural parameters of predicted stable *Imm*2 ArN_{10} at selected pressures.

Phases	Pressure	Lattice Parameters (Å)	Atoms	Wyckoff Positions (fractional) *x*	*y*	*z*
***Imm*2 ArN_{10}**	**100 GPa**	a = 5.02150	Ar1(2b)	-0.50000	0.00000	0.46384
		b = 6.90550	N1(8e)	0.26512	-0.33757	0.50083
		c = 3.77650	N2(8e)	0.14193	-0.20560	0.32603
		α = 90.0000	N3(4c)	-0.35539	-0.50000	0.36292
		β = 90.0000				
		γ = 90.0000				

Table S2. Structural parameters of predicted stable *Imm*2 N_{10} at selected pressures.

Phases	Pressure	Lattice Parameters (Å)	Atoms	Wyckoff Positions (fractional) *x*	*y*	*z*
***Imm*2 N_{10}**	**100 GPa**	a = 7.34460	N1(8e)	-0.33591	0.25768	-0.50155
		b = 5.19350	N2(8e)	-0.20704	0.15270	-0.31753
		c = 4.16020	N3(4d)	-0.50000	-0.34481	-0.37835
		α = 90.0000				
		β = 90.0000				
		γ = 90.0000				

Table S3. Atomic Bader effective charge (e) of some of the valence and conduction bands at Γ k-point of ArN_{10}(*Imm*2).

Phase	Atoms form	Charges(e)
ArN_{10}(*Imm*2) @ 0 GPa	N_{10}-ring	-0.0015
ArN_{10}(*Imm*2) @ 0 GPa	Ar	0.0084
ArN_{10}(*Imm*2) @ 100 GPa	N_{10}-ring	-0.0249
ArN_{10}(*Imm*2) @ 100 GPa	Ar	0.0313
Ordinal of nitrogens in N_{10}-ring	Charges(e)	
	0 GPa	100 GPa
1	-0.0639	0.0136
2	-0.0642	0.0133
3	-0.0640	0.0137
4	-0.0639	0.0139
5	0.0396	0.0001
6	0.0397	-0.0002
7	0.0396	-0.0052
8	0.0395	-0.0052
9	0.0483	-0.0356
10	0.0481	-0.0354

Table S4. Calculated gravimetric chemical energy density (E_d), volumetric energy densities (E_v), detonation velocity (V_d), and detonation pressure (P_d) of ArN_{10}. Compared to the experimental values of the known TNT and HMX explosives. The superscript *expt* and *cal* represent the experimental data and our calculation.

Compound	E_d (kJ/g)	E_v (kJ/cm^3)	V_d (km/s)	P_d (kbar)
ArN_{10}	9.1	25.33	17.56	1712
N_{10}	12.3	36.11	22.35	2832
TNT^{cal}	1.6	6.32	6.76	189
TNT^{expt}	1.6	7.05	6.90	190
HMX^{cal}	3.4	6.46	10.1	470
HMX^{expt}	5.7	10.83	9.10	393

ArN_{10}(vasp)

```
   1.000000
    -2.5107486740837079     3.4527276464763492     1.8882378158545805
     2.5107486740837079    -3.4527276464763492     1.8882378158545805
     2.5107486740837079     3.4527276464763492    -1.8882378158545805
   Ar   N
     1    10
DIRECT
  0.4638408307378353   0.9638408307378352   0.5000000000000000   Ar1
  0.8383998314848202   0.2357091926139875   0.0724457185721609   N1
  0.1632634740418336   0.7659541129126521   0.9275542814278390   N2
  0.1632634740418336   0.2357091926139875   0.3973093611291745   N3
  0.8383998314848202   0.7659541129126521   0.6026906388708257   N4
  0.5316262718104758   0.1840973805326328   0.0636692956085078   N5
  0.1204280849241321   0.4679569762019609   0.9363307043914921   N6
```

0.1204280849241321	0.1840973805326328	0.6524711087221713	N7
0.5316262718104758	0.4679569762019609	0.3475288912778288	N8
0.8629224223698417	0.7183171753425818	0.8553947529727397	N9
0.8629224223698417	0.0075276693970951	0.1446052470272602	N10

N_{10}(vasp)

1.000000

-2.5967224602134569	3.6722878554829923	2.0801189354515626
2.5967336103077296	-3.6722814985250007	2.0801217238394694
2.5967621492644866	3.6723099484457622	-2.0801072243750443

N

10

DIRECT

0.8374550637576235	0.2438678418538842	0.0782271725324506	N1
0.1656386924285727	0.7592258824127427	0.9217698405469505	N2
0.1656395023991593	0.2438703561896673	0.4064127666200008	N3
0.8374552104750492	0.7592205467775131	0.5935863388805416	N4
0.5245690403053231	0.1648283350747874	0.0543338850472139	N5
0.1104890278320383	0.4702322696544954	0.9456624083659406	N6
0.1104935427921419	0.1648255974025474	0.6402559460377262	N7
0.5245700393724562	0.4702337213379622	0.3597461033845448	N8
0.8783511497920813	0.7231622903646624	0.8448119861690202	N9
0.8783474035997898	0.0335418316859091	0.1551935524156107	N10

Reference

1. Wang, Y.; Lv, J.; Zhu, L.; Ma, Y. CALYPSO: A method for crystal structure prediction. *Comput. Phys. Commun.* **2012**, *183*, 2063–2070.
2. Wang, Y.; Lv, J.; Zhu, L.; Ma, Y. Crystal structure prediction via particle-swarm optimization. *Phys. Rev. B—Condens. Matter Mater. Phys.* **2010**, *82*, 094116.
3. Perdew, J.P.; Burke, K.; Ernzerhof, M. Generalized gradient approximation made simple. *Phys. Rev. Lett.* **1996**, *77*, 3865.
4. Lee, I.-H.; Martin, R.M. Applications of the generalized-gradient approximation to atoms, clusters, and solids. *Phys. Rev. B* **1997**, *56*, 7197.
5. Kresse, G.; Furthmüller, J. Efficiency of ab-initio total energy calculations for metals and semiconductors using a plane-wave basis set. *Comput. Mater. Sci.* **1996**, *6*, 15–50.
6. Blöchl, P.E. Projector augmented-wave method. *Phys. Rev. B* **1994**, *50*, 17953.
7. Pack, J.D.; Monkhorst, H.J. "Special points for Brillouin-zone integrations"—A reply. *Phys. Rev. B* **1977**, *16*, 1748.
8. Parlinski, K.; Li, Z.; Kawazoe, Y. First-principles determination of the soft mode in cubic ZrO_2. *Phys. Rev. Lett.* **1997**, *78*, 4063.
9. Ceperley, D.M.; Alder, B.J. Ground state of the electron gas by a stochastic method. *Phys. Rev. Lett.* **1980**, *45*, 566.
10. Grimme, S.; Ehrlich, S.; Goerigk, L. Effect of the damping function in dispersion corrected density functional theory. *J. Comput. Chem.* **2011**, *32*, 1456–1465.
11. Parrinello, M.; Rahman, A. Crystal structure and pair potentials: A molecular-dynamics study. *Phys. Rev. Lett.* **1980**, *45*, 1196.
12. Becke, A.D.; Edgecombe, K.E. A simple measure of electron localization in atomic and molecular systems. *J. Chem. Phys.* **1990**, *92*, 5397–5403.
13. Dronskowski, R.; Blöchl, P.E. Crystal orbital Hamilton populations (COHP): Energy-resolved visualization of chemical bonding in solids based on density-functional calculations. *J. Phys. Chem.* **1993**, *97*, 8617–8624.
14. Maintz, S.; Deringer, V.L.; Tchougréeff, A.L.; Dronskowski, R. *LOBSTER: A Tool to Extract Chemical Bonding from Plane-Wave Based DFT*; Wiley Online Library: Hoboken, NJ, USA, 2016.

15. Johnson, E.R.; Keinan, S.; Mori-Sánchez, P.; Contreras-García, J.; Cohen, A.J.; Yang, W. Revealing noncovalent interactions. *J. Am. Chem. Soc.* **2010**, *132*, 6498–6506.
16. Otero-de-la-Roza, A.; Johnson, E.R.; Luaña, V. Critic2: A program for real-space analysis of quantum chemical interactions in solids. *Comput. Phys. Commun.* **2014**, *185*, 1007–1018.
17. Humphrey, W.; Dalke, A.; Schulten, K. VMD: Visual molecular dynamics. *J. Mol. Graph.* **1996**, *14*, 33–38.
